# “Having vaccines is good but not enough”: Requirements for optimal COVID-19 immunization program in Vietnam

**DOI:** 10.3389/fpubh.2023.1137401

**Published:** 2023-03-09

**Authors:** Linh Phuong Doan, Nam Gia Dao, Duy Cao Nguyen, Trang Huyen Thi Dang, Giang Thu Vu, Long Hoang Nguyen, Linh Gia Vu, Huong Thi Le, Carl A. Latkin, Cyrus S. H. Ho, Roger C. M. Ho

**Affiliations:** ^1^Institute for Global Health Innovations, Duy Tan University, Da Nang, Vietnam; ^2^Faculty of Medicine, Duy Tan University, Da Nang, Vietnam; ^3^Institute of Health Economics and Technology (iHEAT), Hanoi, Vietnam; ^4^National Centre for Youth Substance Use Research, The University of Queensland, Brisbane, QLD, Australia; ^5^Department of Public Health Sciences, Karolinska Institutet, Stockholm, Sweden; ^6^Institute for Preventive Medicine and Public Health, Hanoi Medical University, Hanoi, Vietnam; ^7^Bloomberg School of Public Health, Johns Hopkins University, Baltimore, MD, United States; ^8^Department of Psychological Medicine, Yong Loo Lin School of Medicine, National University of Singapore, Singapore, Singapore; ^9^Institute for Health Innovation and Technology (iHealthtech), National University of Singapore, Singapore, Singapore

**Keywords:** COVID-19, vaccine, allocation, delivery, Vietnam

## Introduction

Vaccination has certainly played the deciding role in easing the deadly grip of the COVID-19 pandemic on people all over the world. The unprecedented swiftness of vaccine development and approval has made this crucial solution available to the humankind at a critical time point, however, efficient and timely delivery of the vaccine to each needed individual has been what ensures the effectiveness of vaccination achieved. In this regard, each country needs to maintain an optimal strategy to allocate and deliver vaccines based on pandemic characteristics and the number of vaccines they have purchased and received at specific time points.

The preparation of Vietnam for the COVID-19 vaccination program can be considered a case study for similar settings, as the country has managed to implement an efficient vaccination program that boost the vaccination rate from 7.5% of adults having a least one dose to 100% in 5 months from August 2021 to February 2022 (1). What is more, this unprecedented vaccination campaign was administrated in the period when COVID-19 was at its worse in Vietnam as the Delta variant spread, racking up cases and claiming life, putting a heavy burden on the healthcare system. The comprehensive preparation plan made by the government of Vietnam early on as soon as the vaccination delivery support from COVAX was announced (2, 3) thus has been the key to the success of Vietnam's COVID-19 vaccination program. This plan has enabled the country to build on its existing immunization infrastructure, which proved to be effective in assisting vaccination programs for other diseases (4), with the option for major expansion when needed.

## Legislation, policy, and plan development and promulgation

From the beginning of 2021, to ensure the legality to facilitate the reception and use of vaccines, the Vietnam Government issued a series of policies, plans, and guidelines for the reception, storage, distribution, and use of COVID-19 vaccines, including Decision No. 1210/QD-BYT dated February 9, 2021, of the Ministry of Health for approving the Plan of receipt, storage, distribution and use of COVID-19 vaccines between 2021 and 2022 supported by the COVAX Facility; Decision No. 1464/QD-BYT dated March 5, 2021, of the Ministry of Health promulgating instructions on receipt, preservation, distribution, and use of COVID-19 vaccine; Decision No. 1467/QD-BYT dated March 5, 2021, of the Ministry of Health for approving the Plan for vaccination against COVID-19 from 2021 through 2022. Each policy had specific regulations on the resources mobilization and allocation, identification of priority groups in the immunization program, as well as implementation plans to achieve herb immunity by 2022. With these policies, each locality when receiving COVID-19 vaccines would be able to develop a clear and transparent vaccine distribution plan, as well as provide vaccines to those who need them during this phase.

## Identification of the vaccinated population

Based on the Recommendation of the WHO Strategic Advisory Group of Experts (SAGE) on immunization, the proposal of the National Immunization Technical Advisory Group (NITAG) for Use of Vaccines and Medical Biologicals of the Ministry of Health at the meeting on November 16, 2020, current COVID-19 epidemic prevention strategy in Vietnam as well as the guidance on surveillance, isolation, and response to COVID-19 epidemic in Vietnam, target groups that need COVID-19 vaccination were sorted by level priority based on epidemic situations and limited availability of vaccine in Vietnam (Table 1) (3). Concerning geographical areas for vaccination, priority would be given according to a level of infection or disease-spreading risk in the areas, including areas with reported morbidity and/ or mortality of community cases of COVID-19, large urban centers with high population density, and provinces with important traffic hubs.

**Table 1:** Vaccination target groups according to the schedule of vaccine supply.

**Target group**	**Level of priority**	**Expected time to be vaccinated**
**- Medical staff. - Staff participating in epidemic prevention (Steering committee at all levels, staff of the quarantine areas, reporters…)**.	**1st: to get vaccinated when the first batch of vaccine received**	**Q1 and Q2 of 2021**
- Diplomatic staff, customs officers, and immigration officers. - Army. - Police force. - Teacher.	2nd: to get vaccinated when the 1st level priority group has reached the expected number of successfully vaccinated	Q1 and Q2 of 2021 (after the 1^st^ level group)
- The general population, with priority given to the following groups: - People over 65 years old. - Group of essential service providers: aviation, transport, tourism; provided electricity and water services ... - People with chronic diseases. - Persons wishing to go to work, study or work abroad. - People in epidemic zones according to epidemiological indications	3rd: to get vaccinated when the 2nd level priority group has reached the expected number of successfully vaccinated	Starting Q3 of 2021 (subject to vaccine delivery by WHO)

To date, based on the safety, immunogenicity, and protection effectiveness, nine vaccines had been approved for emergency use in Vietnam, according to the Ministry of Health, include: AstraZeneca, Sputnik V, Comirnaty (known as Pfizer BioNTech), Vero-Cell, Spikevax (known as Moderna), Janssen, Hayat-Vax, Abdala and Covaxin (Table 2).

**Table 2:** Description of COVID-19 vaccines approved in Vietnam.

**No**.	**Vaccine**	**Origin**	**Manufacturer**	**Approval**	**Note**
1	AstraZeneca (AZD1222)	United Kingdom, Sweden	AstraZeneca	01/02/2021 (5)	2 doses, 8–12 weeks apart
2	Sputnik V	Russia	Gamaleya, Russia	23/03/2021 (6)	2 doses, 3 weeks apart
3	Vero-Cell	China	Beijing Institute of Biologiccal Products Co., Ltd.—China National Biotec Group, China	03/06/2021 (7)	2 doses, 3–4 weeks apart
4	Comirnaty (known as Pfizer BioNTech)	Germany, United States	BioNTech Manufacturing GmbH, Germany	12/06/2021 (8)	2 doses, 3–4 weeks apart
5	Spikevax (known as Moderna)	United States	Moderna	29/06/2021 (9)	2 doses, 4 weeks apart
6	Janssen	Belgium, Netherlands, United States	Janssen Pharmaceutica NV, Belgium and Janssen Biologics B.V, Netherlands	15/07/2021 (10)	1 dose
7	Hayat-Vax	China, UAE	Beijing Institute of Biologiccal Products Co., Ltd.—China National Biotec Group, China; Julphar (Gulf Pharmaceutial Industries)—UAE (Packaged and shipped)	10/09/2021 (11)	2 doses, 2–4 weeks apart
8	Abdala	Cuba	AICA Laboraries, Base Business Unit (BBU), AICA—Cuba	17/09/2021 (11, 12)	3 doses, 14 days apart
9	Covaxin	India	Bharat Biotech International Limited, India	10/11/2021 (11, 12)	

## Transportation

The system of receiving, storing, and distributing vaccines under the Expanded Program on Immunization (EPI) in Vietnam has been deployed through four routes, including national, regional; provincial; and district. The commune level mainly receives vaccines from the district level and deploys vaccination for immunized subjects on the day of vaccination. Communes in the region with remote access would be equipped with small-capacity refrigerators for the storage of vaccines at the commune level. Usually, vaccines would be imported and stored at national-level warehouses then transported and distributed to provincial warehouses, district warehouses, and commune health stations. The Ministry of Health of Vietnam has collaborated with other ministries to review the procedure of importing and transporting vaccines, make necessary changes and agreements to ensure swift and smooth receipt of imported vaccines at the borders as well transition from national-level warehouses to lower-level storage facilities. Temperature monitoring equipment has been used during the transportation and distribution of vaccines at all levels. The transportation of vaccines from the central to the commune level has been done by the vaccination officer. Training and guidance on the transportation and preservation of vaccines will be provided on time.

## Storage

Vietnam can receive, storing, and distributing COVID-19 vaccines under 2–8°C storage conditions at the central, provincial, and district levels. However, it has been necessary to supplement refrigerators for communes in remote areas, with an expected number of at least 2,197 units (of refrigerators) needed. For vaccine storage equipment from −25°C to −15°C, the total available capacity was estimated to be about 16 million doses, which can be considered limited and needs to be supplemented. EPI Vietnam has no capacity for storing vaccines at −80°C to −70°C. The private immunization system such as the Vietnam Vaccine Joint Stock Company (VNVC), on the other hand, has the capacity of 3 million doses storage at this temperature. In case the existing cold storage system of expanded vaccination fails to meet the need for transporting and preserving vaccines, the EPI in coordination with the Department of Preventive Medicine and the Drug Administration of Vietnam has proposed to mobilize cold storage systems of the private sector nationwide.

## Human resources for immunization

At all levels, there is a department in charge of EPI and a specialist in charge of vaccination. Currently, human resources involved in the EPI and private immunization system are being continuously trained in immunization and have experience in organizing immunization sessions. However, the COVID-19 vaccine is a new vaccine, so immunization staff should be retrained on vaccine use and follow-up on post-vaccination adverse events as well as on cold storage management and vaccine storage practices. The Ministry of Health of Vietnam has reviewed documents and training guidelines for COVID-19 vaccination and vaccine preservation of the World Health Organization to develop training plans and materials on COVID-19 vaccination, follow-up of post-vaccination adverse events for vaccination, and preservation facilities. Training on the use of COVID-19 vaccines and post-vaccination adverse event monitoring for vaccination and preservation facilities has been planned to take place before receipt of the supported vaccine.

## Communication

A communication plan on the use of the COVID-19 vaccine has been developed to raise awareness, share information and mobilize people, and mobilize society to participate in vaccination. Meanwhile, messages, reports, and communication materials about the injected subjects as well as information about the COVID-19 vaccine have also been part of the communication plan devised by the Ministry of Health of Vietnam in collaboration with national and private media outlets in the country. Health workers and communicators would be trained in communicating the topics of injection (target group) and information on the COVID-19 vaccine. In addition, information on social networks and in the community on the use of the COVID-19 vaccine would be collected timely and frequently to promptly make plans to overcome and respond to the communication crisis of vaccination. Communication activities according to the approved communication plan would be carried out, specifically providing information to press agencies and communicating to the public about priority subjects, vaccine benefits, injection schedule, the safety of vaccines, post-injection adverse events, and implementation plan. These communication activities would be frequently monitored and supported by the Ministry of Health.

## Organization of vaccination sessions

COVID-19 vaccination would be organized in the form of a vaccination campaign to minimize the delay. The existing expanded immunization system would be utilized while the Department of Health of provinces and cities stands ready to mobilize public and private immunization service establishments to participate in organizing the vaccination session if and when necessary. Guidelines for organizing injection sessions include the organization of vaccination according to the current regulations, the arrangement of injection sites to ensure the prevention of the COVID-19 epidemic and ensure the safety of immunization staff, for proactive monitoring of post-vaccination adverse events and adverse events that require special attention would swiftly be developed and deployed to those involved. As COVID-19 vaccines are new, the monitoring and management of adverse events following vaccination are crucial. Along with on-site monitoring of reactions and events post-vaccination, periodic monitoring in which the immunization facilities conduct follow-up and monitor common reactions and serious complications after injection according to regulations has also been required to be implemented. In addition, advisory councils for assessing the causes of severe catastrophes in the use of vaccines from the central level to the provincial level have been established, organized, and operated. The importance of organizational and educational strategies should be considered. Governments or medical authorities should be prepared to convey to the public in a timely way the potential individual implications of vaccine-related bad situations in order to reassure the public, promote trust in vaccination, and minimize vaccine reluctance (13, 14).

## Epidemiological changes over the implementation of COVID-19 vaccination and antiviral treatment

**Figure 1:**
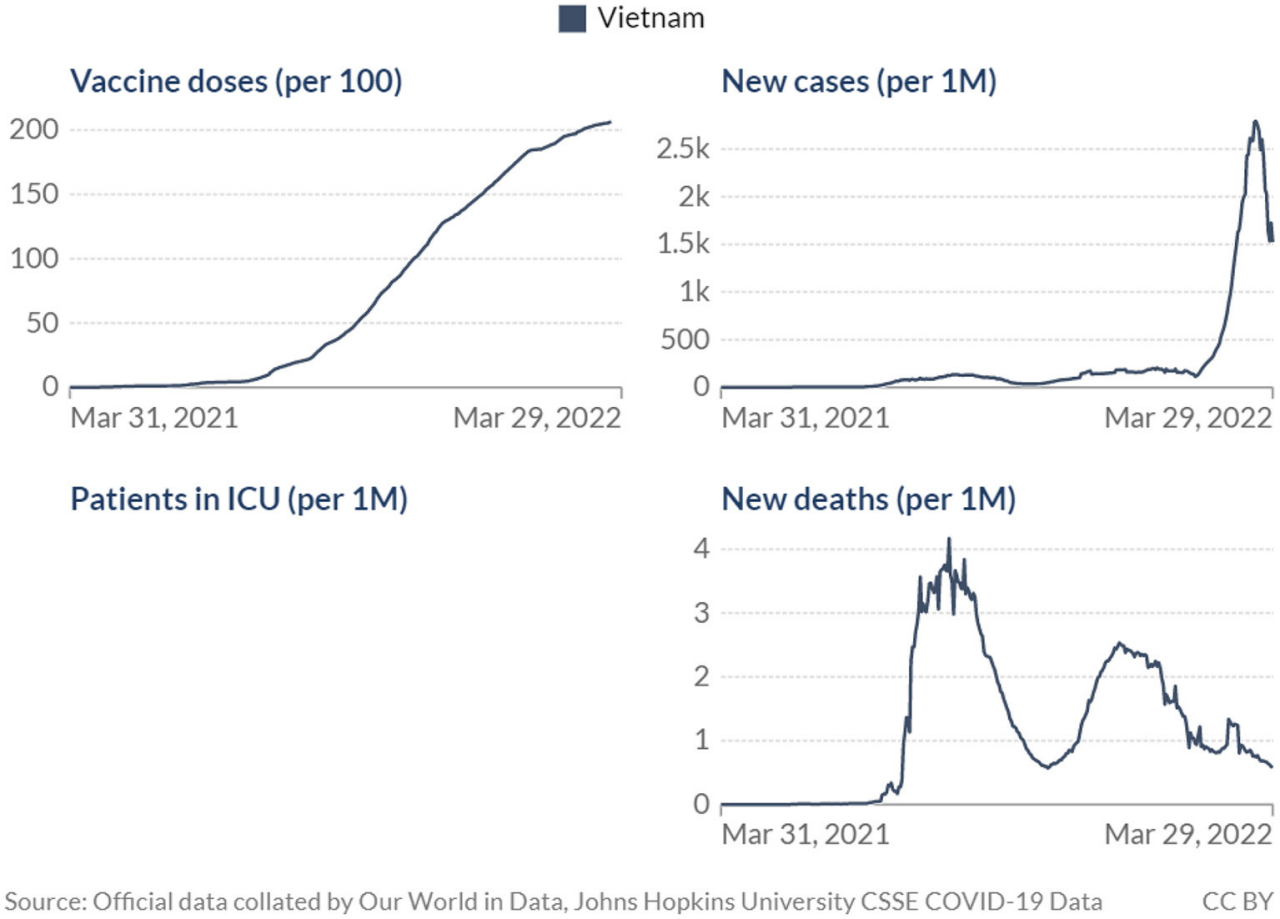
COVID-19 vaccine doses, ICU patients, and confirmed deaths.

Although the impact of the COVID-19 vaccination and antiviral treatment program in Vietnam has not been formally evaluated, observational data have shown some likely outcomes at the population level including the decreased number of new cases and deaths (Figure 1).

It should be noted that the use of COVID-19 antiviral treatment, approved by the government of Vietnam in the last quarter of 2021 (15) would also contribute to the decrease in COVID-19 mortality and severe cases reported above. On the other hand, there could be a time lag between when the policy was implemented and when impacts can be observed *via* health outcomes at the population level (16), thus the effects of the vaccination program may take longer to materialize and be captured in data of cases and deaths. Nevertheless, it is perhaps more important to look beyond the debate of how effective vaccination has been in combating the pandemic. COVID-19 vaccines have been proved time and again, in the lab and in real life, to be effective against the disease, however, concerns have also been raised on the duration of such effectiveness and the role of COVID-19 vaccination in the post-COVID era (17, 18). The data from Vietnam showed decreasing mortality and infection yet worries remained as the number of cases has still been high and the death figures fluctuated significantly. COVID-19 vaccination thus may need to be treated as an ongoing effort rather than a one-off campaign, at least until mortality reaches a constantly low level. Maintaining an immunization plan that would ensure efficiency and be ready to combat any unusual disease-specific issues that may arise would then be crucial going forward. This underlines the role of operations and management in the vaccination program. Understanding the importance of each component of the vaccination plan and how to develop and implement them would be helpful, especially in more resource-scarce settings.

## Conclusion

The arrival of COVID-19 vaccines has been considered the hope for the world in the fight against the deadly disease. Efficient distribution of those vaccines is essential to realize such hope, not only to have the pandemic under control but also to maintain the safety of people in the face of new virus variants and waning vaccine efficacy. Having and maintaining a comprehensive and clear vaccination plan is thus crucial, especially for less advantaged nations. In the case of Vietnam, having a working immunization system in place is important, but even more so is ensuring the involvement of all organizations within the government at national and local levels as well as across all sectors, along with vaccination centers, importers, and distributors from the private side. In addition, it is also noteworthy that, notwithstanding the promises of vaccines, those remedies must be used in conjunction with other tools that have been used so far to prevent the disease's spread and save lives. Working public health measures of mask-wearing, handwashing, social distancing, contact tracing, and other methods must be continued to be implemented.

## Author contributions

Conceptualization: LD, GV, HL, and CL. Methodology: ND, DN, TD, LN, LV, and CL. Supervision: HL, CH, and RH. Writing—original draft: LD, ND, DN, TD, GV, LN, LV, CL, CH, and RH. Writing—review and editing: LD, ND, DN, TD, GV, LN, LV, HL, CH, and RH. All authors contributed to the article and approved the submitted version.
